# The last generation of bacterial growth in limiting nutrient

**DOI:** 10.1186/1752-0509-7-27

**Published:** 2013-03-25

**Authors:** Anat Bren, Yuval Hart, Erez Dekel, Daniel Koster, Uri Alon

**Affiliations:** 1 Department of Molecular Cell Biology, Weizmann Institute of Science, Rehovot, Israel

**Keywords:** Bacterial growth, Monod law, Gene expression, Nutrient limitation, Nitrogen assimilation

## Abstract

**Background:**

Bacterial growth as a function of nutrients has been studied for decades, but is still not fully understood. In particular, the growth laws under dynamically changing environments have been difficult to explore, because of the rapidly changing conditions. Here, we address this challenge by means of a robotic assay and measure bacterial growth rate, promoter activity and substrate level at high temporal resolution across the entire growth curve in batch culture. As a model system, we study *E. coli* growing under nitrogen or carbon limitation, and explore the dynamics in the last generation of growth where nutrient levels can drop rapidly.

**Results:**

We find that growth stops abruptly under limiting nitrogen or carbon, but slows gradually when nutrients are not limiting. By measuring growth rate at a 3 min time resolution, and inferring the instantaneous substrate level, s, we find that the reduction in growth rate μ under nutrient limitation follows Monod’s law, $\mu =\mu_{0}\frac{s}{k_{s}+s}$. By following promoter activity of different genes we found that the abrupt stop of growth under nitrogen or carbon limitation is accompanied by a pulse-like up-regulation of the expression of genes in the relevant nutrient assimilation pathways. We further find that sharp stop of growth is conditional on the presence of regulatory proteins in the assimilation pathway.

**Conclusions:**

The observed sharp stop of growth accompanied by a pulsed expression of assimilation genes allows bacteria to compensate for the drop in nutrients, suggesting a strategy used by the cells to prolong exponential growth under limiting substrate.

## Background

In recent years there has been a resurge of interest in bacterial growth laws, a field of study initiated over 60 years ago but not yet fully understood [1-4]. Studies on microbial growth kinetics were initiated by the seminal studies of Monod [5,6] who measured the relation between sugar concentration and bacterial growth rate. For high sugar levels, where the substrate concentration was in excess, Monod directly measured the exponential growth rate. At low initial sugar levels, that declined during growth due to bacterial consumption, Monod was unable to measure the substrate levels directly, and relied on the assumption that bacteria grow with a constant yield – that is, a unit increase in biomass corresponds to a constant times a unit decrease in sugar concentration. Therefore, the total generated biomass in a density interval along the growth curve allows one to infer the amount of substrate utilized. Monod found a simple mathematical relation connecting the bacterial growth rate μ to the concentration of a growth-limiting substrate s, known as the Monod law:

(1) $$\mu =\mu_{0}\frac{s}{k_{s}+s}$$

where μ_0_ is the growth rate at saturating substrate, and k_s_ is the substrate level at which growth rate is half maximal [6]. Many subsequent theoretical and experimental studies with several substrates and different bacteria found similar growth laws, and suggested several modifications to this law [7-13].

Most of these studies on growth laws measured bacteria in a steady-state situation, using a chemostat or balanced growth in a batch culture inoculated at high dilution [6,9,10,14]. Measuring growth laws in dynamical situations - such as batch cultures which deplete substrate and enter the stationary phase [5] - is much more complicated. A highly dynamical situation occurs in the transition phase between exponential growth and stationary phase where growth stops (also called the deceleration phase). This period, in which μ is strongly influenced by s, is brief, making it difficult to analyze [3,11], both in terms of substrate-growth relations, and gene expression: most studies on gene expression lack the temporal resolution to address such rapidly changing situations [15-18].

Here, we study the last stages of growth in limiting nutrient, in which nutrient levels drop dramatically. To achieve this, we use a robotic assay that allows for the measurement of bacterial growth and gene expression at high temporal resolution (~3 min). We also calculate the concentration of the limiting substrate during growth using Monod’s constant yield assumption. We used *E. coli* grown on limiting concentrations of ammonia or glucose as model systems and found that when the bacteria run out of substrate, growth stops abruptly. In the last generation prior to this abrupt stop in growth, bacteria increase the activity of the promoters of metabolic genes in the pathways that utilize the nutrient in a pulse like manner. Consequently, bacteria can maintain their maximal growth rate during this last generation. When the promoter activity reaches its maximal level, growth rate drops in a way that fits Monod law. Genetic perturbations that abolish this pulse of gene expression alter the way that cells decelerate growth, turning an abrupt stop into a gradual one.

## Results

### High resolution dynamic measurements of growth rate under nutrient limiting conditions

We measured the growth curve of *E. coli* grown on M9 minimal medium supplemented with different amounts of nitrogen in the form of ammonia (NH_4_Cl) ranging from a severely limiting level (0.2 mM), to saturating level (18.7 mM). Optical density in 96-well plates was measured every 3 min, in a robotic system which moved the plate between an incubator (37°C) and an automated fluorimeter. Each plate contained two conditions, with 48 replicates each. Averaging over the 48 repeats yields a standard error in OD on the order of 2% at each time-point. Experiments were repeated 2–5 times with a day-day reproducibility error of 7%.

**Figure 1:**
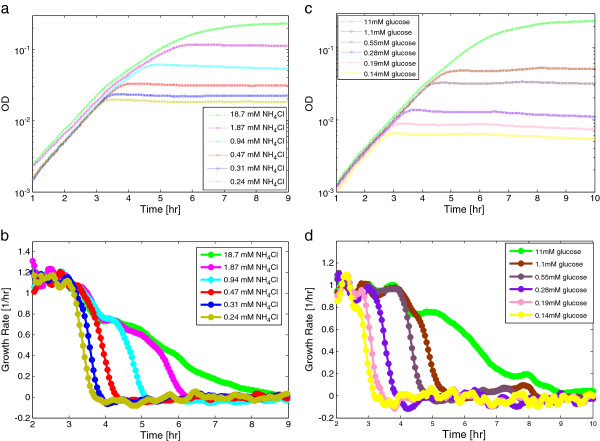
**Under nitrogen or glucose limitation growth rate declines in a sharp manner.***E. coli* was grown in M9 minimal medium with the indicated concentrations of nitrogen (in the form of NH_4_Cl) (a+b) or glucose (c+d). Panels **a**+**c** show OD measurements (600 nm) at a time resolutions of 3 min under nitrogen or glucose limitation respectively. Each point in the graph represents the average OD of 48 experimental replicas with standard error on the order of ~2% at each time-point. Panels **b**+**d** show the growth rate (1/hr) of each OD curve respectively (growth rate is the logarithmic derivative of the OD signal).

The exponential growth rate was very similar at all nitrogen levels (generation time of 59 ± 4 min). We found that for limiting nitrogen levels, the cells grow exponentially and then abruptly stop growth. The lower the nitrogen level, the earlier growth stopped and the lower the final OD level (Figure 1a). Thus, a limiting level of a nutrient in the context of this study means a level which does not support the final OD obtained for the saturating nutrient level, rather than a level which reduces growth rate.

We further analyzed the declaration phase of growth, experiments that are enabled by the 3 min temporal resolution of our assay. We find that at low nitrogen levels, cells stop growth abruptly, going from maximal to zero growth within 27 ± 4 min (Figure 1b, olive green and blue lines). Such an abrupt stop of growth on limiting ammonia levels was previously reported qualitatively [19,20]. At the highest nitrogen levels, 18.7 mM – at which nitrogen is not limiting - cells slow growth gradually as they enter stationary phase [21]. This gradual drop lasts about 4 h (Figure 1b, green line). At intermediate nitrogen levels, cells show a switch between these two behaviors: they first slow growth gradually, and then abruptly stop (see for example Figure 1b, cyan line, Additional file 1: Figure S5).

We find similar results for glucose as a limiting substrate. Cells grown on M9 minimal medium with ample nitrogen (18.7 mM NH_4_Cl) and low levels of glucose (less than ~0.5 mM) stop growth abruptly, going from maximal growth rate to zero growth within 30 ± 3 min (Figure 1d, yellow, pink and purple lines). An abrupt stop of growth in glucose limitation for *E. coli* was previously observed qualitatively [19,20]. At high glucose levels (more than 11 mM), growth slows gradually over about 4 h (Figure 1d green line). At intermediate glucose levels cells show a transition between gradual slowing and abrupt stop of growth (Figure 1d brown line).

### Decline in growth rate as a function of substrate level is well described by the Monod equation

**Figure 2:**
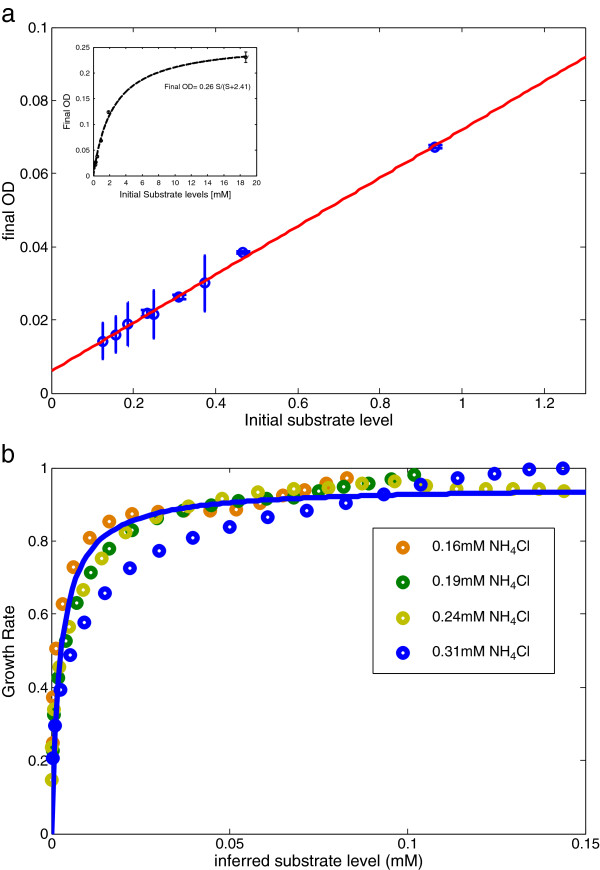
**Under nutrient limitation decline in growth rate is well described by Monod equation.** (**a**) Final OD correlates linearly with initial substrate (NH_4_Cl) level: OD_f_(s) = a*s + b, with a = 0.066 ± 0.003 and b = 0.006 ± 0.001. This relation only saturates at high (more than 2 mM) initial nitrogen level (inset). The intersect of the linear graph is not zero probably due to low levels of nitrogen which were transferred to the media from the over-night culture together with the bacterial inoculum. (**b**) Growth rate as a function of substrate level, calculated using the linear relation between OD and substrate levels in (**a**). Line: fit to Monod law, with K_s_ = 2.6 ± 0.4 μM. The 0.31 mM curve deviates from the Monod fit more than other curves for unknown reasons.

The present assay allows estimation of the substrate level at each time point. Instead of a direct measurement of the substrate level, which is challenging to perform at high temporal resolution and accuracy at low substrate levels, we inferred the substrate level from the bacterial density. To do this, we assume, that the substrate removed from the medium by the cells is incorporated into their biomass with a constant yield [5] ; and that the total biomass (or cell volume) produced is proportional to the OD, as previously demonstrated [10,22]. Indeed, we found that as long as the substrate is limiting, the final OD reached by the culture is proportional to the initial substrate level (Figure 2a). Since under-limiting conditions the final OD is significantly lower than the maximal OD (reached by the non-limited culture) it is unlikely that growth is limited by factors other than the limiting nutrient (that is, effects of the bacteria on the medium other than depletion of the limiting nutrient can be safely neglected due to the low bacterial concentration). The relation between final OD and initial substrate level only begins to saturate when the substrate become non-limiting (initial NH_4_Cl concentrations higher than about 2 mM (Figure 2a inset)). The slope of the proportionality line, c, allows one to translate OD units into substrate units. In this way we calculate the substrate at each time point s(t), using the OD reached at time t:

(2) $$s\left(t\right)=s\left(t=0\right)-\mathrm{cOD}\left(t\right)$$

Next, we plotted the observed growth rate as a function of the inferred substrate at each time point. We found that for low substrate media – in which growth stops abruptly - the decline in growth rate in the deceleration phase is well described by the Monod law, with K_s_ = 2.6 ± 0.4 μM for nitrogen (Figure 2b). Similar results are found for glucose, with K_s_ = 5 ± 1 μM (Additional file 1: Figure S1).

The K_s_ values estimated here can be compared to those estimated in steady-state exponential growth. To our knowledge, the value of K_s_ for nitrogen has not been previously reported. The value for glucose lies within the large range of previously measured K_s_ which spans almost 3 orders of magnitude (from ~0.5 mM to 0.4 μM, [11,13,23,24]). These large differences were attributed to strain variations, differences in growth methods, bacterial density, length of exposure to low glucose concentrations, or the history of the inoculi [1,2,11].

### Promoter activity of nitrogen and carbon assimilation genes rises sharply in the last generation before growth stops

The present assay allows measuring, along with the growth rate at each moment, the promoter activity of selected genes. For this purpose, we used reporter strains in which the promoter of interest controls the expression of a green fluorescent protein (GFP). Reporter strains were taken from a comprehensive library of *E. coli* reporters, in which promoters control the expression of the fast folding and highly stable GFPmut2 [25,26]. We studied the dynamic expression from selected promoters at a time resolution of 8 min. Promoter activity is measured by the rate of accumulation of green fluorescence per OD unit as described [25,27].

**Figure 3:**
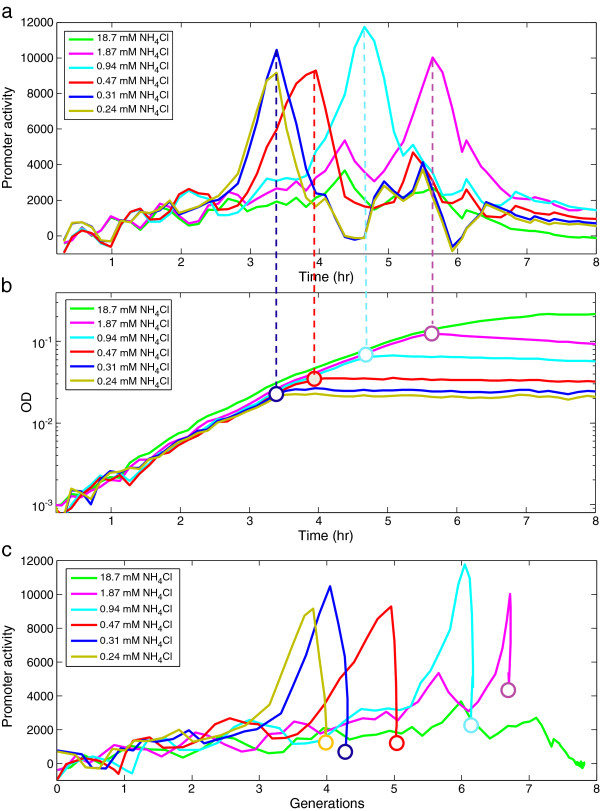
***glnA *promoter is sharply activated in the last generation of growth.** (**a**) Promoter activity of *glnA* as a function of time at several levels of initial nitrogen with a time resolution of 8 min. Promoter activity was calculated by computing the rate of accumulation of GFP per unit time divided by the OD (dGFP/dt/OD). Each point in the graph represents the average promoter activity of 48 experimental replicas with standard error on the order of ~3% at each time-point (**b**) OD curves for the same experiment, with the position of the peak in promoter activity overlaid on the growth curve (open dots). (**c**) same as (**a**) where the x axis is generations instead of time.

We studied the *glnA* promoter which controls an operon of genes essential for ammonia assimilation (glutamine synthetase *glnA*, the nitrogen regulator *ntrC* and its regulatory partner kinase *ntrB*[28-30]). We find that under nitrogen limiting conditions the *glnA* promoter during exponential phase had moderate activity that is independent of nitrogen levels. Then, about one generation before growth stopped, promoter activity rose sharply by about 4–6 fold (Figure 3). Promoter activity dropped back to low levels when growth stopped. The level of nitrogen at which the rise occurs is about the same, 0.25 ± 0.04 mM, for the three lowest nitrogen levels tested (Figure 3).

In contrast to the pulse of activity at the end of growth on limiting nitrogen, *glnA* promoter activity remained roughly constant throughout growth on non-limiting nitrogen (M9 standard formula, 18.7 mM NH_4_Cl), and it gradually declined during entry to stationary phase (green line in Figure 3). We also tested the *glnK* promoter which controls other genes involved in ammonia assimilation (the nitrogen regulatory protein *glnK*, and the ammonia transporter-*amtB *[31-33]). We found very similar results: a sharp rise of promoter activity one generation before growth halts on limiting nitrogen, and a basal level that is nitrogen independent during exponential growth (not shown). This is consistent with previous experiments by Ninfa and colleagues who showed that *glnA* and *glnK* are activated when *E. coli* is starved for ammonia [18]. In contrast, non-related promoters (*e.g*. *crp* and sigma70 synthetic promoters [34], *clpD, serA, cysD* promoters) showed no increase in promoter activity at the end of growth under nitrogen limiting conditions (not shown). Moreover, the abrupt stop in growth upon nitrogen limitation is not accompanied by increased activity of promoters controlling known stationary phase genes (*e.g*. *wrbA, uspB*, as well as a sigmaS synthetic reporter, not shown)[35].

The strong promoter activity peaks and shut-down follow a hill-like function (Additional file 1: Figure S6) reminiscent to previous experimental and theoretical studies which found bi-stability and positive feedback loops in the nitrogen assimilation enzymes and transporters under very low nitrogen levels [36,37].

The rise in *glnA* promoter activity in the last generation before growth stops at low nitrogen, is consistent with previous findings that under severely limiting nitrogen (0.19 mM of NH_4_Cl), *glnA* expressed from a regulated promoter must be over-expressed by 4–5 fold over its wild-type basal levels (at high nitrogen) in order to attain the same growth rate [38]. In that experiment, *glnA* was deleted from the chromosome and placed under control of a *tac* promoter. Thus, the pulsed expression of *glnA* in the last generation of growth is prevented. We tested this mutant strain in the present system, and found that it showed a gradual reduction in growth rate, rather than a sharp stop, at limiting nitrogen levels and low induction levels (Additional file 1: Figure S2). At high induction of *glnA*, the abrupt stop is restored (Additional file 1: Figure S2).

**Figure 4:**
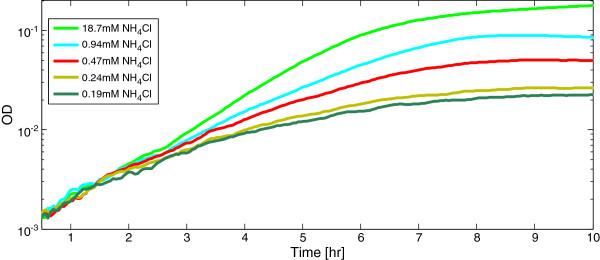
**In a strain deleted for the regulator NtrC growth rate declines gradually.***E. coli ΔntrC* was grown in M9 minimal medium with the indicated concentrations of nitrogen (in the form of NH_4_Cl). OD (600 nm) was measured at a time resolutions of 3 min. Each point in the graph represents the average OD of 48 experimental replicates.

We also tested the effect of removing NtrC- a transcriptional regulator of the *glnA* operon- as well as other operons involved in nitrogen metabolism [28]. An *ntrC* deletion strain which is defective in the regulation of nitrogen metabolic genes showed slower growth rate at low nitrogen levels, and gradual rather than abrupt stop of growth (Figure 4). This finding indicates that transcription regulation by NtrC is essential in order to obtain maximal growth rate and an abrupt stop of growth under nitrogen limiting conditions.

Similar results are also found on limiting glucose (Additional file 1: Figure S3). Here, we studied a reporter for the activity of CRP, a central regulator of sugar metabolism. The reporter plasmid contains a consensus site for CRP controlling GFP expression [34]. We find that CRP activity is moderate and glucose-level-independent throughout exponential growth (4 first hours of growth, Additional file 1: Figure S3). In glucose limitation it shows a rise of about 3-5-fold that lasts about one half of a generation, before growth stops (Additional file 1: Figure S3). In non-limiting glucose (11 mM), CRP activity rises gradually, remaining high in early stationary phase (Additional file 1: Figure S3). Similar results were also found for the *ptsG* promoter, which controls the expression of the PtsG subunit of the PTS glucose permease [39,40] (Additional file 1: Figure S4). It should be noted that in this case the promoter is highly active also in the non-limiting conditions since glucose is the sole carbon source but the increase and decline in promoter activity is moderate compared to the limiting conditions. This observation is in line with recent findings showing a pulse of cAMP level and a sharp increase in the promoter activity of the *acs* gene upon glucose exhaustion [41].

Taken together, these results suggest that up regulation of the relevant metabolic genes in the last generation of growth allows prolonged exponential growth followed by a sharp decline in growth. The pulse of metabolic proteins at the last generation may compensate for the sharp decline in substrate in this phase of growth.

## Discussion

We used a robotic assay to measure bacterial growth rate, substrate level and promoter activity at high temporal resolution across the growth curve. We find that growth stops abruptly under limiting nitrogen or carbon but slows gradually when these nutrients are not limiting. The abrupt stop is accompanied by a pulse-like up regulation of the expression of genes in the relevant nutrient assimilation pathways. Disrupting the regulation of these genes abolishes the pulse of expression, and turns the sharp stop of growth into a gradual deceleration. Reduction in growth rate under nutrient limitation follows Monod’s law, evaluated at each moment with the instantaneous level of substrate.

Bacterial growth laws have mostly been measured in exponential phase in batch culture or in chemostats (see [3,11] for reviews). Studies of growth dependence on substrate in dynamical situations are scarce due to lack of experimental methods that can accurately probe such situations [5]. The present assay enables measurement of growth laws in a batch culture, including the stages where substrate is rapidly depleted by cells nearing stationary phase. The measurements were enabled by the high temporal resolution of the robotic assay, and the large number of repeats which allowed growth to be measured with a standard error of about 2%. Difficulties in measuring very low substrate concentrations are by passed by using accurate measurements of OD and a calibration curve relating OD (biomass) to substrate [13] - a method that can in principle be generalized to other substrates that are incorporated into biomass. Using this approach we could add many experimental points to Monod’s original data on glucose limitation and extend it to nitrogen limitation. In both cases we found that Monod equation fits the data well.

**Figure 5:**
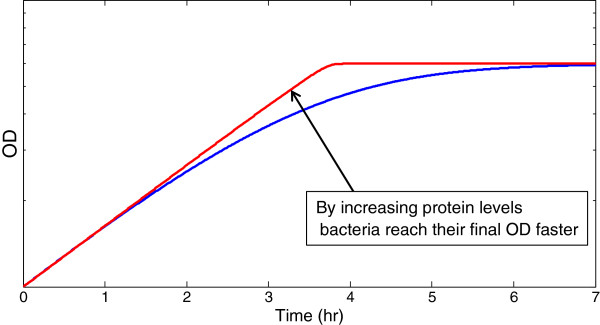
**By increasing the levels of assimilating proteins bacteria reach their final OD faster.** A schematic representation for a mechanism for rapid growth in the last generation. A pulse of metabolic proteins allows the cell to compensate for a sharp decline in substrate. This allows continued exponential growth at about the same speed as maximal exponential growth rate. Growth ends abruptly when substrate goes below the Monod constant. If the pulse of metabolic enzymes is prevented (eg. by mutating the regulators), a gradual stop of growth is obtained. In a hypothetical competition between the wild-type and mutant strain, the wild-type would deplete resources faster and outgrow the mutant in the last generation. Over repeats of this competition, the wild-type strain would be selected. This is a basis for the potential selective advantage of the regulatory strategy found in the present study.

The results suggest a mechanism used by the cells to prolong exponential growth under limiting substrate. The cells express a low basal level of assimilation proteins throughout exponential growth (this level is independent of substrate levels). Then, when substrate drops below a critical level (about 0.25 mM in the case of nitrogen in the form of NH_4_Cl), the cells up regulate the enzymes, regulators and transporters in the assimilation pathway. Such maximal regulator activity only at extreme signal is consistent with the finding that positive feedback regulation in the Pho system is active only at very low signal levels [42] . In our system the pulse of expression allows cells to maintain their rapid exponential growth rate for about one more generation (Figure 5). In this generation, they are able to utilize the remaining substrate. In other words, instead of growth rate declining at 0.25 mM nitrogen, the enzymes allow rapid growth until about 100-fold lower nitrogen levels, on the order of Ks = 2.6 ± 0.4 µM. Growth stops when substrate drops below Ks.

## Conclusions

The present study extends our understanding of the last generation of bacterial growth in batch culture prior to entry into stationary phase. We find that growth stops abruptly under limiting nitrogen or carbon and that reduction in growth rate follows Monod’s law. By following promoter activity of different genes we found that the abrupt stop of growth is accompanied by a pulse-like up-regulation of the expression of genes in the relevant nutrient assimilation pathways. This mechanism allows the cells to maintain their growth rate for about one more generation in which they are able to utilize low levels of substrate. The results presented in this study suggest a strategy used by the cells to prolong exponential growth under limiting substrate.

## Methods

### Strains and plasmids

All strains in this study were derivatives of NCM3722 strain, a standard wild-type *E. coli* strain used in studies of the nitrogen system [43]. For growth rate measurements we used the NCM3722 parental strain. For measuring promoter activity we used plasmids from our comprehensive library of reporter strains. In this library promoter of interest controls a green fluorescent protein gene (GFP) optimized for bacteria (gfpmut2) on a low copy plasmid (pSC101 origin) [25]. For the current study we transformed several selected reporter plasmids to NCM3722. *ΔntrC* strain was obtained by transducing the deletion from the Keio knockout collection (derived from the BW25113 strain, [44]) into NCM3722 by P1 transduction.

### Growth rate and promoter activity measurements

We explored changes in growth rate along the growth curve by high resolution and accurate measurements of the average growth rate of 48 cultures in a 96-well plate using a robotic system [38,45]. Cells were grown overnight in M9 minimal medium (42 mM Na_2_HPO_4_, 22 mM KH_2_PO_4_, 8.5 mM NaCl, 18.7 mM NH_4_Cl, 2 mM MgSO_4_, 0.1 mM CaCl) containing 11 mM glucose, and 0.05% casamino at 37°C to ensure non limiting conditions for the pre-culture. For the nitrogen limitation assay 96-well-plates were prepared using a robotic liquid handler (FreedomEvo, Tecan) with 150 μl of M9 minimal medium containing different levels of NH_4_Cl ranging from 0.16 mM to 18.7 mM and 11 mM glucose (without casamino) (each plate contained two different levels of NH_4_Cl with 48 replicates arranged in a checkerboard format). For the glucose limiting assay M9 minimal medium was used with different levels of glucose ranging from 0.14 mM to 11 mM arranged in wells according to a checkerboard pattern. The wells were inoculated with bacteria at a 1:500 dilution from the overnight culture. This high dilution likely eliminates most of the nutrients leftovers from the overnight culture. Wells were covered with 100 μl of mineral oil (Sigma) to prevent evaporation, a step which we previously found not to significantly affect growth [27,46], and transferred into an automated incubator. Each experiment included 3 plates, allowing measurements of the growth rates in 6 different nutrient concentrations. Cells were grown in an automated incubator with shaking (6 hz) at 37°C for about 20 hours. Every 3 minutes the plate was transferred by a robotic arm into a multi-well fluorimeter (Infinite F200, Tecan) that read the bacteria optical density (OD, 600 nm). Growth rate was calculated for the entire growth curve between successive OD measurements and averaged for the 48 replicates in each condition. Day-to-day relative error in growth rate was 7%. For promoter activity measurements we used the same experimental platform with selected reporter strains. The medium in this case also contained also 50 μg/ml of kanamycine, and in addition to OD measurements we also measured the GFP fluorescence (535 nm) of the cultures. In this case the time resolution of measurements was ~8 min. Promoter activity was calculated by computing the rate of accumulation of GFP fluorescence per unit time divided by the OD (dGFP/dt/OD) as described [27].

### Calculation of substrate levels

A conversion ratio between OD and substrate levels was computed using linear regression at different limiting substrate conditions (0.94 mM, 0.47 mM, 0.31 mM, 0.24 mM of NH_4_Cl (see Figure 2)). Substrate curves were then calculated by: s(t) = s(0) − c OD(t), with c = 15.1 ± 0.7 for nitrogen and c = 21.3 ± 6.3 for glucose. This method relies on the assumption that conversion rate of substrate to biomass (the yield factor) is constant over the timeframe investigated, and that OD is linear in biomass.

## Abbreviations

CRP: cAMP receptor protein; GFP: Green fluorescent protein; OD: Optical density; PA: Promoter activity

## Competing interests

The authors declare that they have no competing interests.

## Authors’ contributions

AB conceived and designed the research, performed the molecular genetics manipulations and the experiments, analyzed data and wrote the paper. YH designed the research, analyzed the data and wrote the paper. ED participated in the design of the study and contributed to the mathematical analysis of the data. DK contributed to the design of the study and helped to write the paper. UA designed the research, analyzed data and wrote the paper. All authors read and approved the final manuscript.

## Supplementary Material

Additional file 1 The last generation of bacterial growth in limiting nutrient. Click here for file
