# Supplementary material for: The last generation of bacterial growth in limiting nutrient
Source: BMC Syst Biol. 2013 Mar 25;7:27. doi: 10.1186/1752-0509-7-27 (PMC3626568; doi:10.1186/1752-0509-7-27)
Supplement: Additional file 1 — The last generation of bacterial growth in limiting nutrient. [file 1752-0509-7-27-S1.docx]

Supplementary information

**The last generation of bacterial growth in limiting nutrient**

Anat Bren, Yuval Hart, Erez Dekel, Daniel Koster and Uri Alon

**1. Decline in growth rate as a function of glucose level is well described by Monod equation.** As we did for nitrogen, we calculated glucose level at each timepoint using the proportionality line, which allows the translation of OD units into substrate units (SI Fig. 1a). Next, we plotted the observed growth rate as a function of substrate at each time-point. We find that for low initial glucose levels where growth is abruptly stopped the decline in growth rate is well described by a Monod law, with K_s_=5+/-1 µM (SI Fig. 1b).


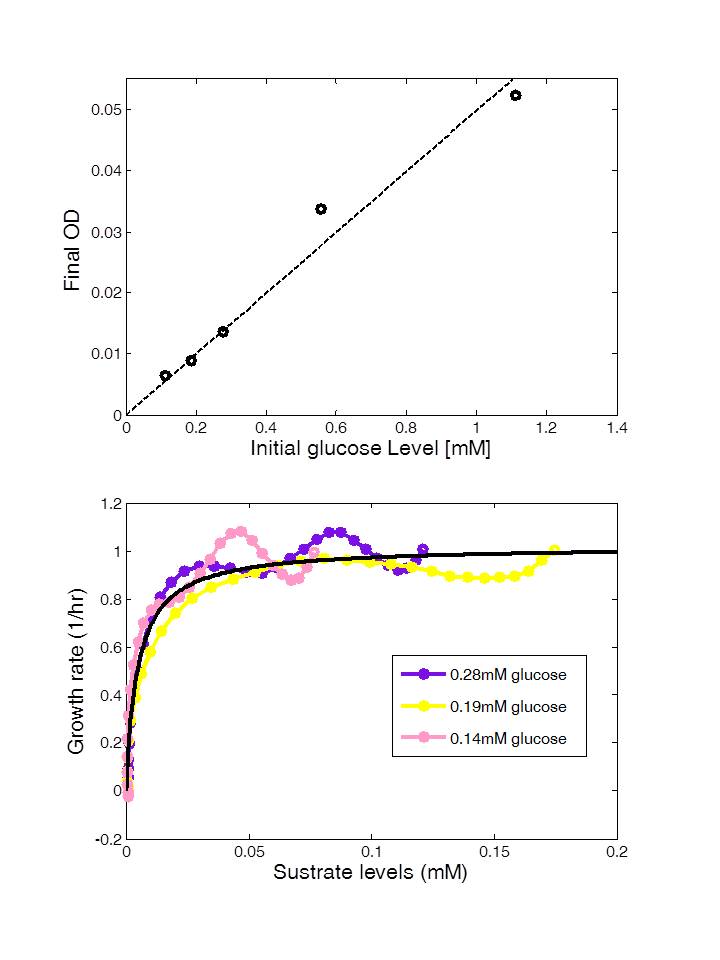


**Fig. 1** (a) Final OD correlates linearly with initial substrate (gluocse) level: OD_f_(s)=a*s+b, with a=0.047+/-0.0016 and b=0. (b) Growth rate as a function of substrate level, calculated using the linear relation between OD and substrate levels in (a). Line: fit to Monod law, with K_s_=5+/-1 µM µM.

**2. Under constitutive expression of *glnA* the decline in growth rate depends on *glnA* levels.** *glnA* in this strain was deleted from the chromosome and expreseed from a plasmid under the control of the *tac* promoter. The cells were grown with limiting levels of nitrogen (0.24mM) and different amount of IPTG reflecting different expression levels [1]. In low expression levels growth rate was low (~0.4 1/hr) and the decline in growth rate lasted more than an hour. With increased *glnA* expression levels which allow a more efficient nitrogen utilization growth rate was higher and it declined faster.
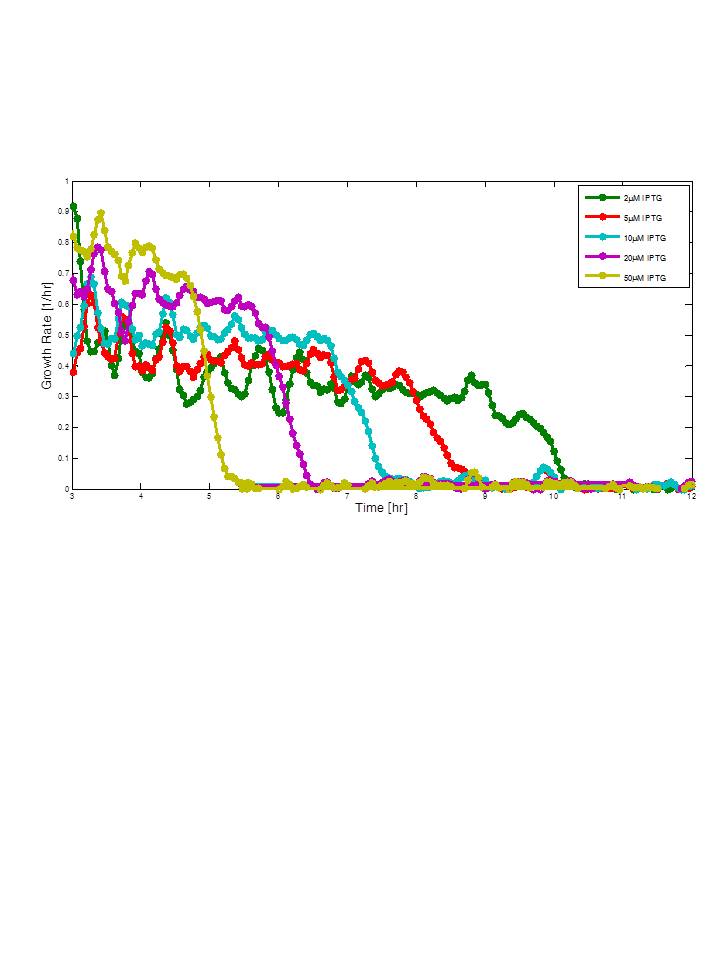


**Fig.2** *E. coli* expresses *glnA* under the regulation of *tac* promoter was grown in M9 minimal medium with 0.24mM nitrogen (in the form of NH_4_Cl) and the indicated concentration of IPTG. Shown is the growth rate (1/hr) obtained for OD measurements at a 3min time resolution (growth rate is the logarithmic derivative of the OD signal).

**3. Promoter activity of a synthetic CRP-regulated promoter rises sharply in the last generation before growth stops on glucose limitation.**

**
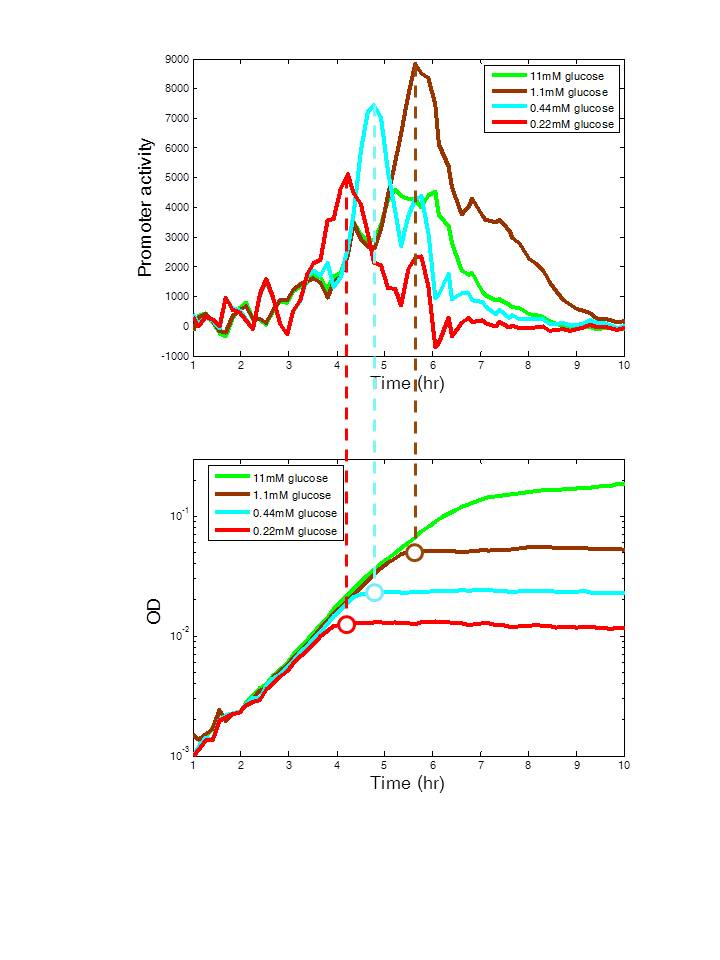
Fig 3**. (a) Promoter activity of a synthetic CRP reporter strains as a function of time at several levels of initial glucose. Promoter activity was calculated by computing the rate of accumulation of GFP per unit time divided by the OD (dGFP/dt/OD). (b) OD curves for the same experiments, with the position of the peak in promoter activity overlayed on the growth curve (open dots).

b

a

**4. Promoter activity of *ptsG-*a subunit of the glucose PTS permease rises sharply in the last generation before growth stops on glucose limitation.**

**Fig. 4** (a) Promoter activity of *ptsG* reporter strain as a function of time at several levels of initial glucose. Promoter activity was calculated by computing the rate of accumulation of GFP per unit time divided by the OD (dGFP/dt/OD). (b) OD curves for the same experiments, with the position of the peak in promoter activity overlayed on the growth curve (open dots).

The *ptsG* promoter is regulated by an incoherent feed forward loop circuit with CRP activating both *ptsG* and its repressor, Mlc. The incoherent FFL has been shown to generate pulses of gene expression experimentally [2-3], and in mathematical models [4]. This is a potential mechanism for pulse generation upon carbon starvation for the *ptsG* gene.

**5. At intermediate nitrogen levels, growth rate decline can be divided to two phases.**

At intermediate nitrogen levels (1.87 and 0.94mM NH_4_Cl) decrease in growth rate shows a transition between gradual decrease which follows the decline under non-limiting conditions and a sharp decline.

**
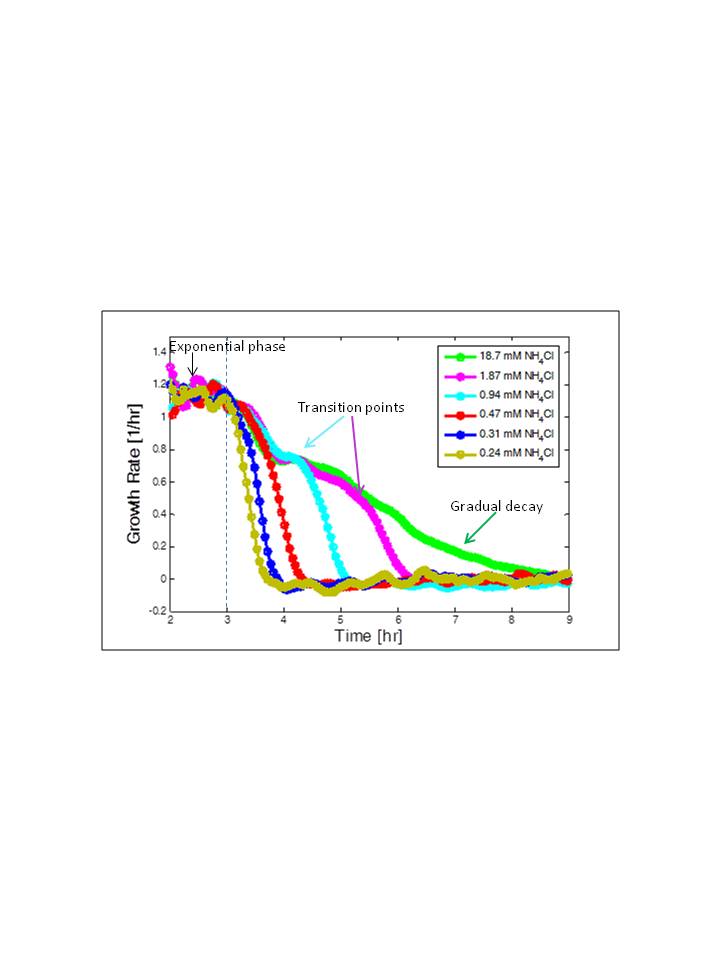
**

**Fig. 5.** **Decline in growth rate at intermediate nitrogen level show transitions between two rates of decline.** *E. coli* was grown in M9 minimal medium with the indicated concentrations of nitrogen (in the form of NH_4_Cl). Growth rate was calculated from OD measurements (600mm) at a time resolution of 3 min.

**6. Promoter-activity dynamics at low nitrogen levels indicate a transition from Michaelis-Menten kinetics to Hill-like kinetics.**

In the following we describe possible models for promoter activity that maintain the optimal growth rate despite depletion of substrate. The crux of all models lay in the ability to exactly compensate substrate depletion by increasing protein numbers (transporters [5] or assimilation enzymes[6]).

Several models can support our observed promoter activity behavior which indicates a sharp pulse in enzyme production during the last generation of bacteria growth. Among these models are hill-like sensing and production of enzymes, two enzymes/transporters system with differing affinities and an Incoherent feedforward loop mechanism (IFFL).

Promoter activity (PA) measurements for low nitrogen levels (0.24-0.93mM) indicate that there is a transition of PA dynamic levels from a simple Michaelis-Menten kinetics for enzyme levels to a higher hill-like curve (Fig.5). This transition can be the product of two possible mechanisms: either a positive feedback loop that starts at low nitrogen levels [7] or a transition to a higher affinity enzyme with action of both enzymes [6]. Below we describe the dynamical equations of both models.

**Fig. 6: Promoter activity shows a transition between Michaelis-Menten kinetics to Hill-like kinetics as initial nitrogen levels decrease.** Measurements of promoter activity normalized to sigma70 promoter activity and plotted versus calculated substrate levels. Promoter activity curves almost coalesce for low nitrogen levels (). Solid lines in the lower panel display fit of the data to a Hill-like curve with a hill coefficient of two (n=2) and Michaelis-Menten curve (n=1) in blue and black respectively.

In our model, substrate (s) effect at low levels is to turn on a positive feedback loop. Then, growth rate dependence on substrate levels is described by a Hill-like curve.

1. $g(t)=g_{0}\frac{s^{n}}{s^{n}+K_{m}^{n}}E$

As bacteria grow, they deplete the substrate s from the medium. Thus, if enzyme levels (E) are constant, growth will slow down. In order to maximize growth, bacteria can increase enzyme levels or activity in order to counter the depletion of substrate. Thus, we assume that enzyme levels are substrate dependent, E(s). To keep a constant, high growth rate, enzyme must increase in a way that exactly cancels the decrease in substrate, in the following form

1. $E(s)=E_{0}\frac{s^{n}+K_{m}^{n}}{s^{n}}$

Plugging equation (2) into equation (1), results in a constant, high growth rate g(t)=g_0_.

However, this strategy cannot be maintained indefinitely, because enzyme levels diverge to infinity according to Eq.(2), when substrate concentration becomes very low (s<<Km).

To address this divergence, cells must stop producing enzyme, and Eq. (2) must be modified:

1. $E(s)=E_{0}\frac{s^{n}+K_{m}^{n}}{s^{n}+K_{s}^{n}}$

This dynamics is very similar to Eq.(2) at high to intermediate substrate levels (s>Ks), but saturates when substrate levels go below Ks. Thus, enzyme levels increase to exactly counter-balance the depletion of substrate until a point where enzyme production reaches high levels that are no longer feasible or beneficial to the bacteria.

From Eq.(3) one can derive a formula for the PA:

1. $PA\left( s \right)=\frac{dE\left( s \right)}{ds}\frac{ds}{dt}+g\left( s \right)E(s)$

which gives the following curve for PA as a function of substrate:

1. $PA\left( s \right)=P_{0}\frac{s^{n}\left( K_{m}^{n}+s^{n} \right)\left( K_{s}^{n}+s^{n} \right)+n\left( K_{m}^{n}-K_{s}^{n} \right)s^{2n-1}}{\begin{aligned} \left( K_{s}^{n}+s^{n} \right)^{3} \\ \end{aligned}}$

At high substrate levels, PA levels are fixed at P_0_. When substrate levels approach K_m_, PA levels rise and when substrate levels approach K_s_ a shut-down of enzyme production occurs. This behavior resembles our experimental results of the pulse in PA levels throughout the depletion process of nitrogen by the cells.

Similar results may occur when two enzymes, E_1_ with low affinity (K_m1_) and E_2_ with high affinity (K_m2_) exist. In this case, growth is described by

1. $g\left( t \right)=g_{0}\left( \frac{s}{s+K_{m1}}E_{1}+\frac{s}{s+K_{m2}}E_{2} \right)=g_{0}\left( \frac{E_{1}s\left( s+K_{m2} \right)+E_{2}s\left( s+K_{m1} \right)}{\left( s+K_{m1} \right)\left( s+K_{m2} \right)} \right)$

We assume that at high substrate levels mainly E_1_ is produced while E_2_ levels are kept constant. As substrate levels decrease towards K_m1_, E_2_ levels should rise in order to activate the higher affinity enzyme while E_1_ levels remain constant. Again, E2 levels should counter-balance the depletion of substrate:

1. $E_{2}\left( s \right)=E_{2,0}\frac{{(K}_{m2}+s)(K_{m1}+s-E_{1}s)}{s(K_{m1}+s)}$

Again, as substrate levels go to zero, enzyme levels diverge. In order to account for this diversion, we take E_2_(s) to be

1. $E_{2}\left( s \right)=E_{2,0}\frac{{(K}_{m2}+s)\left( {(K}_{m1}+s \right)-E_{1}s)}{(s+K_{s})(K_{m1}+s)}$

Where K_s_ represents the cutoff in enzyme levels, in the sense that the ratio $\frac{K_{m2}}{K_{s}}$ is the factor by which E_2_ levels can increase up to s -> 0. Following the same procedure as for the previous case of an effective Hill-like dynamics for growth one has for the PA:

1. $PA\left( s \right)=\frac{g_{0} s \left( K_{s} E_{1}+s+K_{m1} \right)}{\left( s+K_{s} \right)^{3}\left( s+K_{m1} \right)^{3}}{(\left( k_{m1}+s \right)}^{2}\left( s^{2}+K_{m2}\left( K_{s}+2s \right) \right)-E_{1}s^{2}\left( K_{m2}K_{s}+K_{m1}\left( K_{m2}-K_{s} \right)+2K_{m2}s+s^{2} \right))$

While capturing the needed rise in enzyme levels, this model does not capture enzyme production shutdown as substrate levels decrease below Ks.

**References**

1. Hart Y, Madar D, Yuan J, Bren A, Mayo AE, Rabinowitz JD, Alon U: **Robust control of nitrogen assimilation by a bifunctional enzyme in E. coli.** *Mol Cell* 2011, **41:**117-127.

2. Basu S, Mehreja R, Thiberge S, Chen MT, Weiss R: **Spatiotemporal control of gene expression with pulse-generating networks.** *Proc Natl Acad Sci U S A* 2004, **101:**6355-6360.

3. Mangan S, Itzkovitz S, Zaslaver A, Alon U: **The incoherent feed-forward loop accelerates the response-time of the gal system of Escherichia coli.** *J Mol Biol* 2006, **356:**1073-1081.

4. Mangan S, Alon U: **Structure and function of the feed-forward loop network motif.** *Proc Natl Acad Sci U S A* 2003, **100:**11980-11985.

5. Kim M, Zhang Z, Okano H, Yan D, Groisman A, Hwa T: **Need-based activation of ammonium uptake in Escherichia coli.** *Mol Syst Biol* 2012, **8:**616.

6. Muller PJ, von Frommannshausen B, Schutz H: **Regulation of ammonia assimilation in ammonia-limited chemostat cultures of Escherichia coli ML 30: evidence of bistability.** *Z Allg Mikrobiol* 1981, **21:**361-372.

7. Miyashiro T, Goulian M: **High stimulus unmasks positive feedback in an autoregulated bacterial signaling circuit.** *Proc Natl Acad Sci U S A* 2008, **105:**17457-17462.
